# Antiretroviral resistance among HIV-1 patients on first-line therapy attending a comprehensive care clinic in Kenyatta National Hospital, Kenya: a retrospective analysis

**DOI:** 10.11604/pamj.2018.29.186.10796

**Published:** 2018-04-02

**Authors:** Joyceline Gaceri Kinyua, Raphael Wekesa Lihana, Michael Kiptoo, Timothy Muasya, Irene Odera, Patrick Muiruri, Elijah Maritim Songok

**Affiliations:** 1 Kenya Medical Research Institute, Nairobi, Kenya; 2 School of Health Sciences, South Eastern Kenya University, Kenya; 3 College of Health Sciences, University of Nairobi, Kenya

**Keywords:** Drug resistance, mutations, antiretroviral therapy, comprehensive care clinic

## Abstract

**Introduction:**

Antiretroviral therapy plays a major role in reducing the impact of Human Immunodeficiency Virus/Acquired Immune Disease Syndrome, especially in resource-limited settings. However, without proper infrastructure, it has resulted in emergence of drug resistance mutations in infected populations. To determine drug resistance mutations among patients attending a comprehensive care facility in Nairobi, 65 blood samples were successfully sequenced.

**Methods:**

Whole blood samples were also tested for CD4+T-cell count and plasma HIV-1 RNA Viral load. Drug-resistance testing targeting the HIV-1 RT gene was determined. Patients were on first line ART that consisted of two NRTIs, and one NNRTI.

**Results:**

Females were younger (mean 42) than males (mean 45) and lower median CD4+ counts (139 cells/μl) than males (152 cells/μl). The prevalence of drug resistance mutations (any major mutation) in this population was 23.1% (15/65). Major NRTI mutations were detected in 11 patient samples, which included M184V (n = 6), M41L (n=3), D67N (n=2), K219Q (n=3) and T215F (n=2). Major NNRTI mutations were detected in 14 patient samples. They included K103N (n = 10), G190A (n = 1), Y181C (n = 1) and Y188L (n = 1).

**Conclusion:**

Presence of major mutations in this study calls for proper laboratory infrastructure to monitor treatment as well as regular appraisals of available regimens.

## Introduction

Globally, over 34 million people were infected with Human Immunodeficiency Virus (HIV) by the end of 2010 [1]. Ninety percent of them were from resource-limited settings, where there is shortage of Antiretroviral Therapy (ART) [2]. With increasing evidence that treatment programs in resource-limited settings can achieve treatment outcomes comparable to those of developed countries [3-7], most countries in sub-Saharan Africa have implemented policies to increase access to ART [8]. The aim of ART is to suppress HIV replication to below the limit of detection. ART eventually provides the greatest potential for immune reconstitution and minimizes the risk of treatment failure, which arises as a combination of factors, such as bad adherence, interruption of treatment due to drug toxicity, and the emergence of resistance mutations [9, 10].

The first HIV case in Kenya was identified in 1984. Since then, the HIV and Acquired Immune Disease Syndrome (AIDS) epidemic kept rising and remained relatively steady after 2003 with a prevalence of 6.7% among individuals aged 15-49 years (4.6% in men and 8.7% in women). By the end of 2010, it was estimated that 6.3% of Kenyans aged 15-64 years were infected. To improve the life of HIV and Acquired patients and reduce the HIV and AIDS-related morbidity and mortality, the Kenyan government significantly increased access to ART since 2003 [11]. With this increase in ART coverage, the danger of increased drug-resistant strains among drug-naive patients became real. Furthermore, stigma and social backgrounds among the infected population has affected access to ART and compliance, resulting in an accelerated emergence of drug-resistant mutants, especially among those on suboptimal therapy. As in many resource-limited settings, antiretroviral drugs are limited in Kenya and only those meeting some criteria can receive treatment. There is a concern that antiretroviral drug resistance among those on treatment would spread to those acquiring new infections (who may not access treatment) and compromise the current regimens thereby giving rise to early treatment failure among those on ART. This study was carried out with the aim of establishing the prevalence of drug resistance mutations among HIV antiretroviral drugs are limited in Kenya and only those meeting some criteria can receive treatment. There is a concern that antiretroviral drug resistance among those on treatment would spread to those acquiring new infections (who may not access treatment) and compromise the current regimens thereby giving rise to early treatment failure among those on ART. This study was carried out with the aim of establishing the prevalence of drug resistance mutations among HIV-1 infected patients seeking care and treatment from an established government comprehensive care center of Kenyatta National hospital in 2009. This data would assist in better care and service provision to patients so as to improve HIV prevention initiatives for targeted resource allocation and service delivery among the infected.

## Methods

### Study design, subjects and antiretroviral drugs

This was a cross sectional study involving adult patients attending the comprehensive care clinic of Kenyatta national hospital, Kenya in 2009. Demographic data, such as age and gender were obtained through individualized interview. After informed consent and ethical clearance from the Kenyatta Hospital ethical committee, blood samples were collected. The patients were adults on first line antiretroviral medication for at least six months. Those who did not consent were excluded from the study as well as those on 2^nd^ line therapy. First-line antiretroviral drugs consisted of 2 nucleoside reverse transcriptase inhibitors, NRTIs mostly zidovudine (AZT) or tenofovir (TDF) and lamivudine (3TC) or emtricitabine (FTC)] and 1 non-nucleoside reverse transcriptase inhibitor, NNRTI mostly nevirapine (NVP) or efavirenz (EFV)] as stipulated in the world health organization (WHO) guidelines [12].

### CD4+ T-cell counts

CD4+T-cell counts of peripheral blood were determined using the FACSCOUNT (Becton-Dickinson, Beiersdorf, Germany).

### HIV-1 genotyping and drug resistance analysis

HIV-1 RNA was extracted from 100 μl of plasma using SMITEST EX-R and D (Genome Science Laboratories, Fukushima, Japan) according to the manufacturer’s instructions. The HIV-1 reverse transcriptase (RT) gene was amplified by both one step RT polymerase chain reaction (PCR) and nested PCR as previously described [13-16]. The *pol*-RT nucleotide sequences were translated into the corresponding amino acids and analyzed for previously reported drug resistance-associated mutations using the Stanford University HIVdb sequence analysis program [17]. The REGA HIV-1 subtyping tool [18] on the Stanford database was used to determine the HIV-1 subtype of each patient sample based on RT sequences. Generated sequences were aligned using ClustalW and phylogenetic trees viewed using FigTree [19]. Demographic, and immunologic parameters were analyzed using the student’s t test to determine significance (with p value of <0.05).

## Results

### Patient characteristics

Between February and October 2009, 65 patient samples were collected. Of these 39 were from female and 26 from male patients. The mean age was 43 years (42 years for females (SD, 9.67) and 45 years (SD, 8.93) for males). Though males seemed to be older than females, this was not statistically significant (p = 0.2). The average duration on ART was 8 years (8.2 years for females and 7.7 years for males). Median baseline CD4 and viral loads were very low and high for females and males, respectively (Table 1).

**Table 1:** Demographic, virological and immunological characteristics of HIV-1 infected patients attending the Kenyatta National Hospital Comprehensive Care Clinic

	All	Males	Females
Mean Age (years)	43	45	42
Median CD4 [Range] (cell/mm^3^)	149^++^[2-873]	152 [4-873]	139 [2-777]
Median Viral load [Range ] (copies/mm^3^)	75845^+^[475-772940]	125753 [475-750000]	82524 [1643-772940]
Mean duration on ART (years)	8	8.2	7.7
HIV-1 Subtypes			
A1	41	16	25
C	7	2	5
D	16	8	8
G	1	1	0

ART: Antiretroviral therapy;

+ n=13;

++ n=40

### Antiretroviral therapy regimens

The patients were in conventional first line ART as stipulated in Kenyan ART guidelines. However, 21 patients were deemed to be clinically failing their current ART regimens. Most patients were on AZT/3TC/NVP combination at the time of this analysis before changing to AZT/3TC/LPV in their subsequent regimens.

### HIV-1 Drug resistance mutations

Major NRTI mutations were detected in 11 patient samples, which included M184V (n=6), M41L (n=3), D67N (n=2), K219Q (n=3) and T215F (n=2). Major NNRTI mutations were detected in 14 patient samples. They included K103N (n=10), G190A (n=1), Y181C (n=1) and Y188L (n=1) (Table 2). The prevalence of drug resistance mutations in these treatment-experienced patients was estimated to be 26%.

**Table 2:** Characteristics of patients with HIV-1 drug resistance mutations (n = 15)

Sample ID.	Age	Gender	NRTI Mutations	NNRTI Mutations	HIV-1 Subtype
KN13	48	Male	T215F	None	D
KN17	46	Male	K219R	K103N	A1
KN20	40	Male	V75I, M184V	Y181C	A1
KN23	42	Female	None	K103N	D
KN35	48	Female	L210W, T215F	None	A1
KN37	35	Female	T69N, M184V	K103N	C
KN39	39	Male	D67N, K70R, M184V, K219Q	K103N	C
KN40	54	Female	D67N, T69N, K70L, M184V, T215S, K219Q	K101N, Y188L	A1
KN41	46	Male	M41L, V75A	K103N	D
KN47	44	Female	M41L	K103N	D
KN49	39	Female	K70R, M184V, K219Q	K103N	A1
KN54	44	Male	T215P, K219R	G190A	D
KN67	42	Female	None	K103N	D
KN70	39	Male	None	K103N	D
KN78	43	Female	M41L, M184V	K103N	A1

NRTI: Nucleoside reverse transcriptase inhibitor; NNRTI: Non-NRTI

### HIV-1 Subtypes

**Figure 1:**
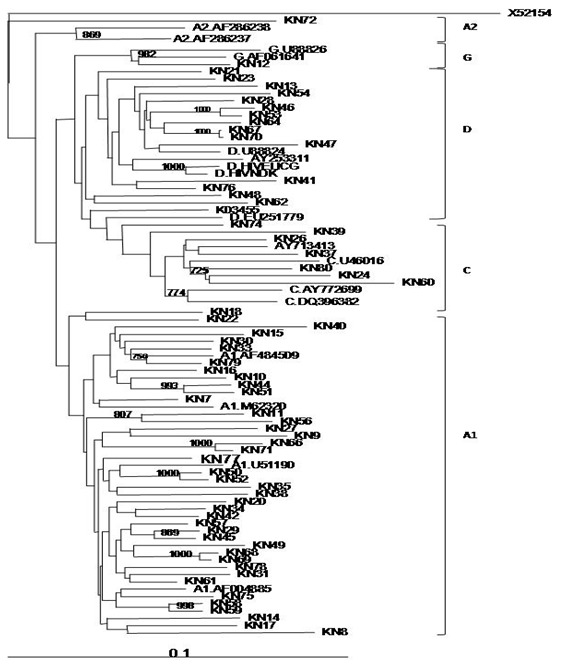
Phylogenetic tree of HIV-1 pol-RT sequences from patients attending the Comprehensive Care Clinic in Kenyatta National Hospital, Kenya

The generated HIV-1 RT sequences, after aligning with reference sequences from the Los Alamos HIV database (using ClustalW software) revealed the following as circulating subtypes in the study population: A1 (41/65, 63.1%), C (7/65, 10.8%), D (16/65, 24.6%) and G (1/65, 1.5%) (Table 1, Figure 1).

## Discussion

In the current study, we analyzed samples from 65 HIV-1 infected patients who were on first-line ART at Kenyatta national hospital comprehensive care clinic for at least eight years. It was shown that the most prevalent HIV-1 subtype among the patients was A1, followed by D and C. As observed from boot scan values in the REGA system, most of these subtypes were pure, representing homogenous strains that are yet to develop recombination (as is the nature of HIV). These findings are in agreement with previous findings in Kenya where subtype A has consistently been reported to be the most prevalent [20, 21].

The prevalence of drug resistance (any major mutation) was estimated to be 23.1% (15/65) among the studied population. This was higher compared to another finding among HIV infected Kenyan Injecting Drug Users where resistance was estimated to be 13.8% [22]. This difference may be attributable to different study populations. The most prevalent NRTI drug resistant mutation was M184V that has been associated with use of lamivudine. Lamivudine forms the backbone of first line ART in Kenya. The most prevalent NNRTI mutation that was detected in the studied population was K103N, which is associated with the use of Nevirapine – also a major component of first line regimens in Kenya. Among the studied patients, follow up was inconsistent for the duration of ART. This translated into missed evaluations of both virologic (viral load) and immunologic (CD4+ counts) parameters that would be used to inform regimen change. As such this might have contributed to emergence of drug resistance. Furthermore, no baseline drug resistance mutation was done before initiation of ART; hence we could not ascertain whether the mutations detected were acquired or transmitted. Similarly, no virologic (viral load) and immunologic (CD4+ counts) data was available at the time of this analysis hence we could not tell which patients were failing treatment in order to advice on regimen change.

In this study, we did bulk sequencing using plasma RNA. However, the limitation of bulk sequencing is that it compromises the detection of minor population of HIV-1 drug-resistant variants, which may exist in low copy numbers due to exposure to ART [23]. Under such circumstances, detection of minor viral variants using more sensitive methods, such as pyrosequencing and clonal or deep sequencing, would be more ideal [24]. Furthermore, our study focused on the HIV-1 RT region only, which is the target of first line drugs. Neither did we do for the protease region (target for second line drugs). This may have underestimated the prevalence of drug resistance in this setting given that baseline drug resistance (both transmitted and acquired) was not done.

## Conclusion

Overall prevalence of drug resistance mutations among CCC patients in KNH in 2009 was 23.1%. This study forms a basis upon which future ART would largely depend in establishing trends so as to guide and inform the establishment of proper laboratory infrastructure to monitor treatment as well as regular appraisals of available regimens.

### What is known about this topic

- Drug resistance is a major impediment to the success of ART in resource limited settings;
- Continued research/surveillance of such is of critical importance to the success of available regimens due to high possibilities of transmitted as well as acquired drug resistance.

### What this study adds

- Information gathered from this study forms a vital component of existing literature on HIV Drug Resistance;
- This information may be valuable when administering regimens that have had a history of either treatment success or drug resistance-associated complications.

## References

[1] United Nations Programme on HIV/AIDS (UNAIDS) (2011). World AIDS day report.

[2] AIDS Info Guidelines for the use of antiretroviral agents in HIV-1-Infected adults and adolescents.

[3] Losina E, Touré H, Uhler LM, Anglaret X, Paltiel AD, Balestre E, Walensky RP, Messou E, Weinstein MC, Dabis F, Freedberg KA (2009). Cost-effectiveness of preventing loss to follow-up in HIV treatment programs: a Côte d'Ivoire appraisal. PLoS Med.

[4] Ciaranello AL, Chang Y, Margulis AV, Bernstein A, Bassett IV, Losina E, Walensky RP (2009). Effectiveness of pediatric antiretroviral therapy in resource-limited settings: a systematic review and meta-analysis. Clinical Infectious Diseases.

[5] Johnston V, Fielding KL, Charalambous S, Churchyard G, Phillips A, Grant AD (2012). Outcomes following virological failure and predictors of switching to second-line antiretroviral therapy in a South African treatment program. Journal of Acquired Immune Deficiency Syndrome.

[6] Phillips AN, Carr A, Neuhaus J, Visnegarwala F, Prineas R, Burman WJ, Williams I, Drummond F, Duprez D, Belloso WH, Goebel FD, Grund B, Hatzakis A, Vera J, Lundgren JD (2008). Interruption of antiretroviral therapy and risk of cardiovascular disease in persons with HIV-1 infection: exploratory analyses from the SMART trial. Antiviral Therapy.

[7] Phillips AN, Pillay D, Miners AH, Bennett DE, Gilks CF, Lundgren JD (2008). Outcomes from monitoring of patients on antiretroviral therapy in resource-limited settings with viral load, CD4 cell count, or clinical observation alone: a computer simulation model. Lancet.

[8] United Nations Programme on HIV/AIDS (UNAIDS) (2010). Global Report: UNAIDS Report on the Global AIDS Epidemic.

[9] Page I, Phillips M, Flegg P, Palmer R (2011). The impact of new national HIV testing guidelines at a district general hospital in an area of high HIV seroprevalence. The Journal of the Royal College of Physicians of Edinburgh.

[10] Cambiano V, Lampe FC, Rodger AJ, Smith CJ, Geretti AM, Lodwick RK, Puradiredja DI, Johnson M, Swaden L, Phillips AN (2010). Long-term trends in adherence to antiretroviral therapy from start of HAART. AIDS.

[11] National AIDS and STI Control Programme, Ministry of Health, Kenya (2012). Kenya AIDS Indicator Survey.

[12] WHO Global HIV/AIDS Response Epidemic update and health sector progress towards Universal Access, Progress Report 2011.

[13] Lihana RW, Lwembe RM, Bi X, Ochieng W, Panikulam A, Palakudy T, Musoke R, Owens M, Ishizaki A, Okoth FA, Songok EM, Ichimura H (2011). Efficient monitoring of HIV-1 vertically infected children in Kenya on first-line antiretroviral therapy. Journal of Clinical Virology.

[14] Lwembe R, Ochieng W, Panikulam A, Mongoina CO, Palakudy T, Koizumi Y, Kageyama S, Yamamoto N, Shioda T, Musoke R, Owens M, Songok EM, Okoth FA, Ichimura H (2007). Anti-retroviral drug resistance-associated mutations among non-subtype B HIV-1-infected Kenyan children with treatment failure. Journal of Medical Virology.

[15] Ndembi N, Abraha A, Pilch H, Ichimura H, Mbanya D, Kaptue L, Salata R, Arts EJ (2008). Molecular characterization of human immunodeficiency virus type 1 (HIV-1) and HIV-2 in Yaounde, Cameroon: evidence of major drug resistance mutations in newly diagnosed patients infected with subtypes other than subtype B. Journal of Clinical Microbiology.

[16] Ndembi N, Hamers RL, Sigaloff KC, Lyagoba F, Magambo B, Nanteza B, Watera C, Kaleebu P, Rinke de Wit TF (2011). Transmitted antiretroviral drug resistance among newly HIV-1 diagnosed young individuals in Kampala. AIDS.

[17] Stanford University HIV drug resistance database.. The HIVdb Program Genotypic Resistance Interpretation Algorithm.

[18] Stanford University HIV drug resistance database.. The REGA HIV-1 Subtyping Tool.

[19] Andrew Rambaut (2007). FigTree - Molecular Evolution, Phylogenetics and Epidemiology.

[20] Land AM, Luo M, Pilon R, Sandstrom P, Embree J, Wachihi C, Kimani J, Plummer FA, Ball TB (2008). High prevalence of genetically similar HIV-1 recombinants among infected sex workers in Nairobi, Kenya. AIDS Research and Human Retroviruses.

[21] Lihana RW, Khamadi SA, Lwembe RM, Kinyua JG, Muriuki JK, Lagat NJ, Okoth FA, Makokha EP, Songok EM (2009). HIV-1 subtype and viral tropism determination for evaluating antiretroviral therapy options: an analysis of archived Kenyan blood samples. BMC Infectious Diseases.

[22] Osman S, Lihana RW, Kibaya RM, Ishizaki A, Bi X, Okoth FA, Ichimura H, Lwembe RM (2012). Diversity of HIV type 1 and drug resistance mutations among injecting drug users in Kenya. AIDS Res Hum Retroviruses.

[23] Nicot F, Saliou A, Raymond S, Sauné K, Dubois M, Massip P, Marchou B, Delobel P, Izopet J (2012). Minority variants associated with resistance to HIV-1 nonnucleoside reverse transcriptase inhibitors during primary infection. Journal of Clinical Virology.

[24] Fisher R, van Zyl GU, Travers SA, Kosakovsky Pond SL, Engelbrech S, Murrell B, Scheffler K, Smith D (2012). Deep sequencing reveals minor protease resistance mutations in patients failing a protease inhibitor regimen. Journal of Virology.

